# Short-term and long-term outcomes of indocyanine green for sentinel lymph node biopsy in early-stage breast cancer

**DOI:** 10.1186/s12957-022-02719-7

**Published:** 2022-08-09

**Authors:** Bin Hua, Yao Li, Xin Yang, Xiaotian Ren, Xu Lu

**Affiliations:** Breast Center, Department of Thyroid-Breast-Hernia Surgery, Department of General Surgery, Beijing Hospital, National Center of Gerontology, Institute of Geriatric Medicine, Chinese Academy of Medical Sciences, Beijing, 100730 People’s Republic of China

**Keywords:** Breast cancer, Indocyanine green, Sentinel lymph node mapping, Safety outcome

## Abstract

**Background:**

Indocyanine green (ICG) is becoming a frequently used sentinel lymph node (SLN) tracer of breast cancer in China*.* However, there is still a lack of data on its safety. We reported the clinical outcome of ICG as a tracer of SLN over a median 67-month follow-up period to evaluate its feasibility in clinically node-negative patients with breast cancer.

**Methods:**

A total of 194 consecutive patients underwent sentinel lymph node biopsy (SLNB) with ICG, radioisotopes (RI) and methylene blue (MB), or with ICG and MB. The SLN mapping data by each tracer was recorded, and safety outcomes were analyzed through follow-up.

**Results:**

With the triad mapping (*N* = 44), the identification rate of SLN by ICG was 95.5%, slightly higher than that of MB (86.4%) and comparable with RI (95.5%) and combined methods (95.5%, 100%) (*p* = 0.068). Analysis of all candidates (*N* = 194) demonstrated that the identification rate of SLN by ICG or by ICG and MB was 99%, significantly higher than that by MB (92.8%) (*p* < 0.0001). No tracer-related allergic reaction and permanent skin staining of ICG were observed. Local disease progression was reported in 2 of the 194 patients at the ipsilateral axilla. After remedial axillary lymph node dissection, no disease progression was detected at follow-up.

**Conclusions:**

ICG as an SLN tracer is more accurate than MB and comparable to the combined methods and has good clinical safety. ICG can be considered a useful supplement or suitable alternative to traditional tracers.

**Supplementary Information:**

The online version contains supplementary material available at 10.1186/s12957-022-02719-7.

## Introduction

Breast cancer is the most common malignant tumor affecting women’s health worldwide and the same in China [1, 2]. Axillary lymph node metastasis is one of the important factors affecting the long-term prognosis and adjuvant therapy decisions of patients with primary breast cancer. Before the 1990s, axillary lymph node dissection (ALND) was the standard method to stage the axillary lymph nodes; however, ALND can cause lymphedema, shoulder joint dysfunction, and other postoperative morbidities, which would affect the quality of life of long-term survivors of breast cancer [3–5]. Therefore, in 1993, after Krag et al. reported the use of gamma-probe localization of radiolabeled lymph nodes to identify the sentinel lymph node (SLN) in breast cancer, sentinel lymph node biopsy (SLNB) was widely considered and gradually accepted by surgeons for application in breast cancer patients with clinically negative axillary lymph nodes [6].

The gold standard for SLN mapping is a radioisotope (RI) combined with blue dye (BD; patent blue, methylene blue (MB), or isosulfan blue) [7–9]. This dual tracer approach achieves a high detection rate (> 90%) and a low false-negative rate (FNR) (< 10%) of SLNs [8]. However, BD can cause allergic reactions and pigmentation of the skin, and when used alone, BD has low sensitivity to locate SLNs [10–12]. RI exposes both the patient and staff to radiation. In addition to the poor timeliness, the RI tracing technique requires professional equipment and mandates special licenses as well as hospital infrastructure for safe use [13–15]. Therefore, it is difficult for RI to be widely carried out worldwide, and the same situation exists in China.

The shortcomings of BD and RI lymphatic mapping have led to the development of new techniques for SLN locations, such as indocyanine green (ICG) fluorescence imaging, superparamagnetic iron oxide nanoparticles, and contrast-enhanced ultrasound using microbubbles [8]. Among them, near-infrared fluorescence SLN mapping with ICG is becoming increasingly popular in China because of its good timeliness, convenient performance, and comparable accuracy with traditional tracers [16].

ICG has been used to trace SLNs in clinically node-negative patients with breast cancer since 2014 in Breast Center of Beijing Hospital. From June 2014 to September 2014, we mapped SLNs with technetium 99-labeled dextran (RI), MB, and ICG to determine the workflow of fluorescent tracing and verify the accuracy of the ICG fluorescent technique. Since October 2014, ICG combined with MB has been routinely used to trace SLNs in our center. Here, we retrospectively report the feasibility and safety outcome of near-infrared fluorescence SLN mapping with ICG.

## Materials and methods

### Ethics and patients

Between June 2014 and December 2015, a total of 194 consecutive patients with clinically node-negative early-stage breast cancer were admitted to the Breast Center of Beijing Hospital and were found suitable to undergo SLNB according to the 2014 ASCO guideline recommendations for sentinel lymph node biopsy in early-stage breast cancer [17].

From June 2014 to September 2014, 44 patients underwent SLNB using the triad-tracer method (RI + MB + ICG). During this process, we confirmed the operating procedure of fluorescent SLN tracing and established a learning curve. Then, from October 2014 to December 2015, 150 patients underwent SLNB by the dual technique (ICG + MB).

The study protocol was approved by the Ethics Committee of Beijing Hospital on the basis of the Declaration of Helsinki (IRB number in ethical approval: 2021BJYYEC-368-01), and written informed consent was obtained from the patients.

### Reagents and equipment

MB (Jumpcan Pharmaceutical Group Co., Ltd., Taixing, China) was used as the blue dye at a final concentration of 2%, and 25 mg of ICG (Dandong Yichuang Pharmaceutical Co., Ltd., Liaoning, China) was diluted at a final concentration of 0.075% according to the previous studies [18–21]. In addition, 185 MBq (5 mCi) RI (HTA Co., Ltd., Beijing, China) was used as the radiotracer. ICG fluorescence was excited and detected by a portable fluorescent vascular imaging system MDM-I (MD Biomedical Technology Corporation, Hebei, China), and the radioisotope signal was detected by an RMD Navigator™ GPS (Gamma Positioning System) (RMD Instruments Corporation, Inc., MA, USA).

### SLNB procedure

RI was injected subcutaneously around the tumor 6–12 h before the operation, and static planar imaging was performed at 1 h and 2.5 h after injection to mark the projection of SLNs on the body surface. For patients undergoing mastectomy, 0.5 ml MB and 1 ml (0.75 mg) ICG were injected intradermally at 1–4 points around the areola 10 min and 5 min before the operation, respectively. For patients undergoing breast-conserving therapy, MB and ICG were injected intradermally around the tumor with a separate incision near the axilla where the fluorescence disappeared or with the same incision of the primary tumor.

In all patients, the near-infrared (NIR) fluorescence signal in the SLN was probe first, and then, radioactive signal from excised lymph nodes was explored, and blue staining was differentiated at the same time; at last, the axillary operation field was detected by Gamma Positioning System or blue dye to find any possible residual SLNs. The mapping patterns of each SLN and the time of SLNB with ICG were recorded in detail.

### Treatment

All resected SLNs were evaluated by intraoperative frozen section analysis in the Pathological Department of Beijing Hospital. Patients with positive SLNs underwent ALND. Otherwise, ALND should be omitted. After the operation, the patients were treated according to the clinical practice guidelines for breast cancer.

### Follow-up and statistical analysis

Follow-up was performed every 3–6 months postoperatively. The deadline was December 31, 2020. The date of breast cancer recurrence, distant metastasis, or new malignant disease in the contralateral breast or other parts of the body (not including skin basal cell carcinoma or cervical intraepithelial neoplasia) was used to calculate disease-free survival (DFS), and the date of death from any cause was used to calculate overall survival (OS). Survival curves were generated using the Kaplan–Meier method. During the follow-up, special attention was given to the first recurrence site in the ipsilateral axilla.

For each tracing technique, the SLN identification rate was equal to the number of successful mappings divided by the total number of mappings performed. The SLN metastasis rate was equal to the number of patients with SLN metastasis divided by the total number of patients undergoing SLNB. The SLNB sensitivity was calculated by dividing the number of patients with SLN metastasis by the total number of patients with axillary lymph node metastasis. FNR was equal to the number of patients with false-negative metastatic SLNs divided by the total number of patients with metastatic axillary lymph nodes. Since ALND was not routinely performed after SLNB according to the 2014 ASCO guidelines [17], the sensitivity and FNR of SLN mapping by various tracers could only be analyzed exploratively.

The Pearson’s chi-squared test or Fisher’s exact test was used to process the categorical variables, and the Kruskal–Wallis test was used to analyze the medians. Two-sided *p*-values < 0.05 were considered to indicate statistically significant differences. IBM-SPSS version 22.0 (IBM Corp., Armonk, NY, USA) and R software version 4.0.5 (R core Team, Vienna, Austria) were used for analysis and image drawing.

## Results

### Clinicopathological data of the enrolled patients

From June 2014 to December 2015, a total of 194 consecutive patients with clinically node-negative early-stage breast cancer were fitted into this retrospective study. After the operation, all patients were routinely followed up in the clinic or by telephone. The clinical and pathological data were retrospectively analyzed and detailed in Table 1.

**Table 1:** Clinical and pathological characters of the enrolled patients

Clinicopathological factors	*N* = 194	%
Age		
≥ 65	64	32.99
< 65	130	67.01
Histological grade		
G1	27	13.92
G2	112	57.73
G3	55	28.35
Estrogen receptor expression		
Positive	155	79.90
Negative	39	20.10
Progesterone receptor expression		
Positive	154	79.38
Negative	40	20.62
HER-2 expression		
Positive	42	21.65
Negative	107	55.15
Unknown	45	23.20
Ki-67 proliferation index		
≥ 14%	133	68.56
< 14%	61	31.44
pTNM stage		
0	5	2.58
I	93	47.94
II	86	44.33
III	10	5.15
Pattern of surgery		
SLNB + M	147	75.77
SLNB + MRM	38	19.59
SLNB +BCT	9	4.64

*BCT* Breast conserving therapy, *HER-*2 Human epidermal growth factor receptor 2, *M* Mastectomy, *MRM* Modified radical mastectomy, *N* Number, *pTNM* Pathological tumor-node metastasis, *SLNB* Sentinel lymph node biopsy

### Fluorescence tracing with ICG was a convenient and accurate technique for SLNB

**Fig. 1:**
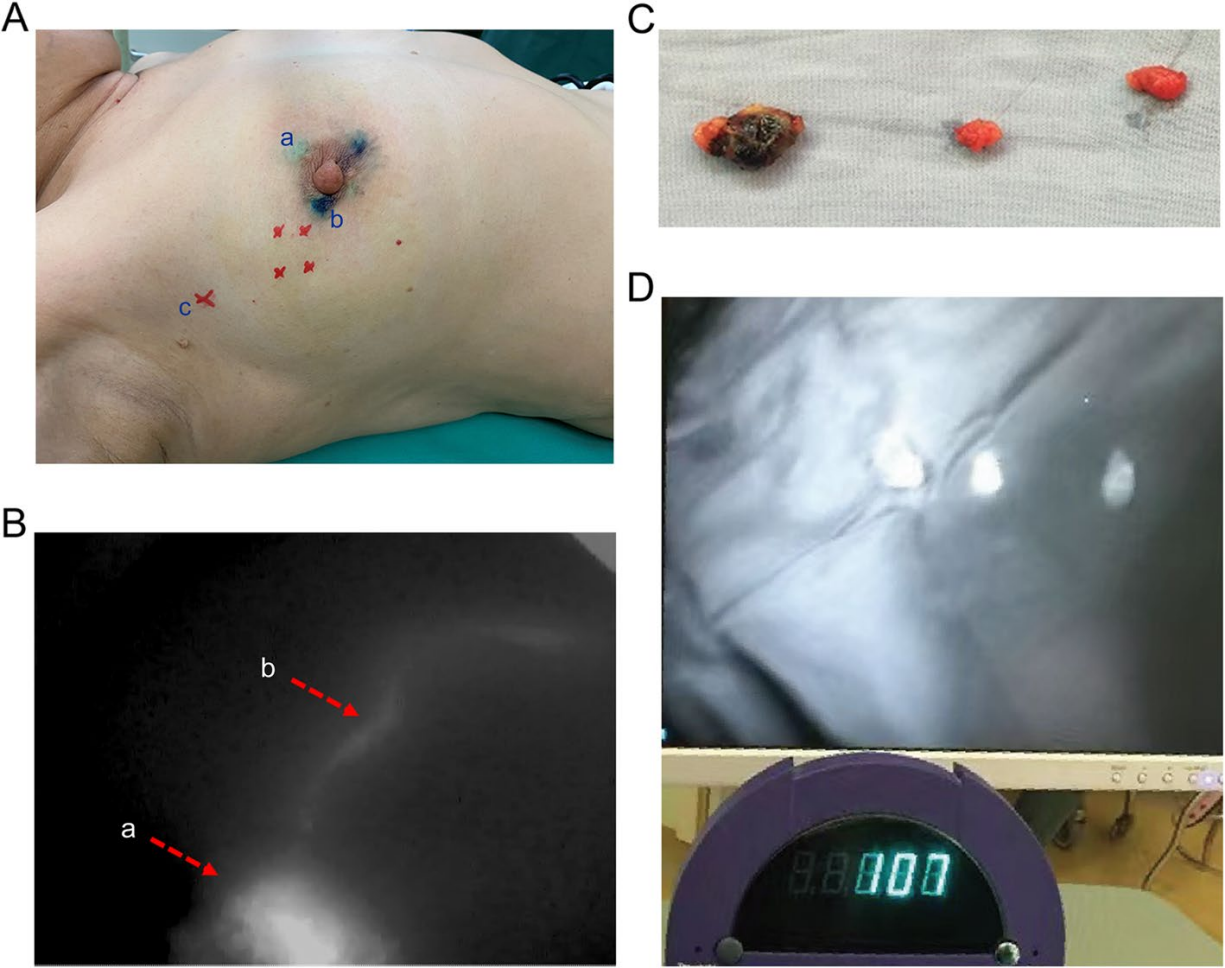
Sentinel lymph node biopsy with indocyanine green, methylene blue and technetium 99-labeled dextran. **A** Points a and b are the intradermal injection points around the areola of indocyanine green (ICG) and methylene blue (MB), respectively, the four red points are the subcutaneous injection points of technetium 99-labeled dextran (RI) around the tumor, and c is the body surface projection of the located sentinel lymph node (SLN) by RI. **B** Fluorescent-positive lymphatic vessel on the body surface can be observed live (arrow a, ICG injected point; arrow b, fluorescent-positive lymphatic vessel). **C** SLNs and the first SLN with blue dye. **D** SLNs with fluorescence and radioactivity detected by the fluorescent vascular imaging system MDM-I and RMD Navigator™ GPS (Gamma Positioning System)

**Fig. 2:**
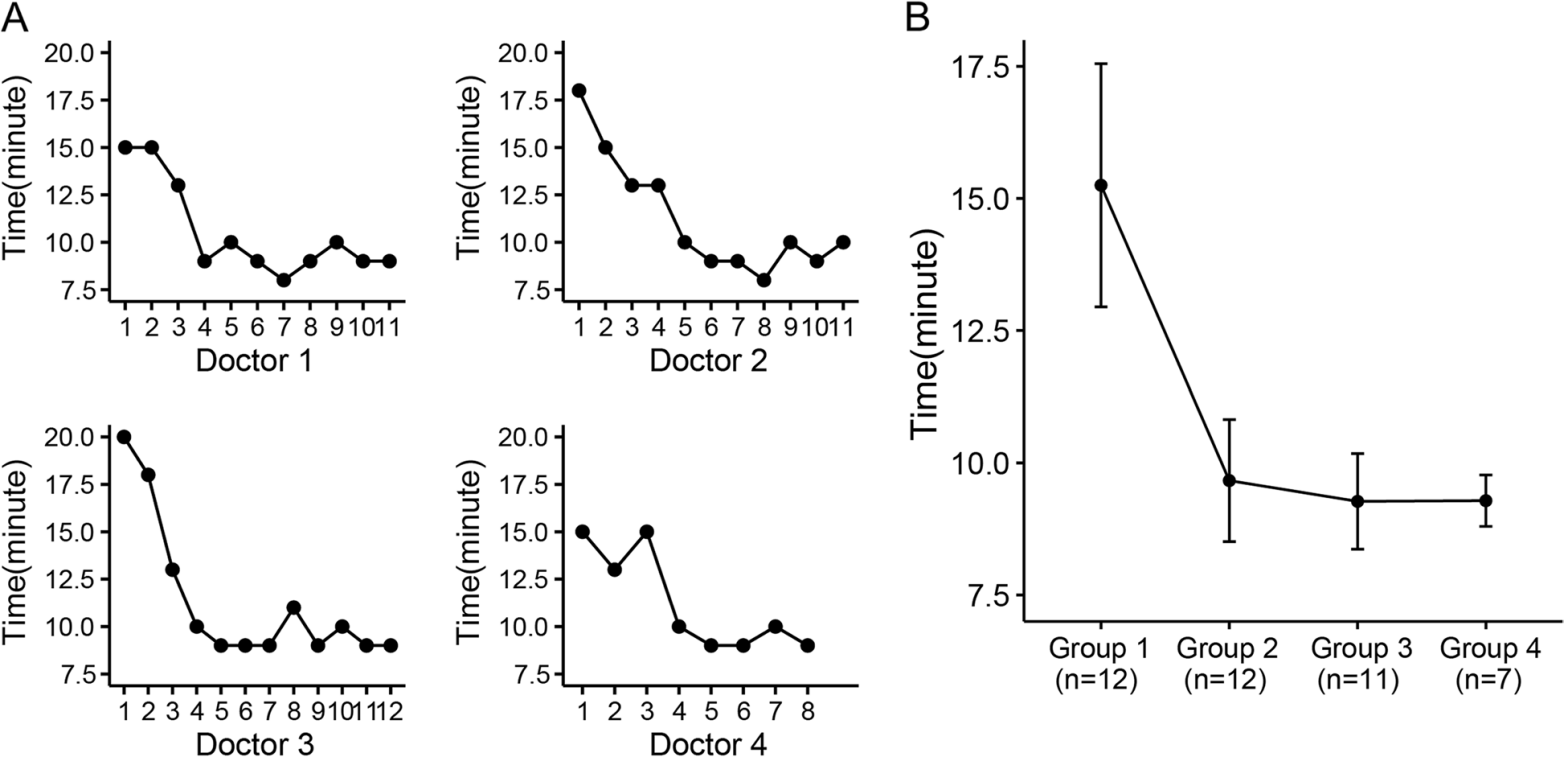
Operation-time learning curve of sentinel lymph node biopsy (SLNB) with indocyanine green (ICG). **A** The operation-time learning curve of one of the four surgeons in our center initially learning to use ICG for tracing SLN. **B** The operation-time learning curve of all four surgeons grouped by every 3 consecutive cases according to the operation order. The time of SLNB with ICG tended to be stable after the fifth operation

From June 2014 to September 2014, SLNB was carried out by triple-tracer method (*N* = 44) (Fig. 1). Four surgeons at our center performed 8–12 SLNBs combined with mastectomy. After they completed the first three SLNBs, the operation time gradually stabilized, and after five operations, the operation time of SLNB with ICG for each surgeon was stable at approximately 10 min (Fig. 2). The median number of SLNs detected by ICG was 3 (interquartile range, *IQR* = 1), which was significantly more than that detected by MB (2(2)) and by RI (2 (1)) (*p* < 0.0001). It was confirmed by triad technique that the accuracy of SLN tracing with ICG was similar to that of the combined method (RI + MB + ICG or ICG + MB) (*p* > 0.05) (Table 2, Supplement Fig.1A).

**Table 2:** SLN fluorescent tracing with ICG was an accurate technique confirmed by triad methods

Parameters	N	ICG	MB	RI	ICG + MB	RI + ICG + MB	*p*
SLN identification rate (%)	44	95.5 (42/44)	86.4 (38/44)	95.5 (42/44)	95.5 (42/44)	100 (44/44)	0.068b
SLN counts (median (IQR))	44	3 (1)	2 (2)	2 (1)	3 (1)	3 (1)	< 0.0001c
False-negative rate (%)	11	0 (0/11)	18.2 (2/11)	18.2 (2/11)	0 (0/11)	0 (0/11)	0.2b
Sensitivity (%)	11	100 (11/11)	81.8 (9/11)	81.8 (9/11)	100 (11//11)	100 (11/11)	0.2b
Metastatic SLN rate (%)	44	25 (11/44)	20.5 (9/44)	20.5 (9/44)	25 (11/44)	25 (11/44)	0.962a
Metastatic SLN counts (median (IQR))	11	2 (4)	1 (1)	1 (1)	2 (4)	2 (4)	0.101c

*ICG* Indocyanine green, *MB* Methylene blue, *N* Number, *RI* Technetium 99-labeled dextran, *SLN* Sentinel lymph node. ^a^By Pearson’s chi-square test, ^b^by Fisher’s exact test, ^c^by Kruskal-Wallis test

From October 2014 to December 2015, SLN was mapped by the dual technique with ICG and MB (*N* = 150). The total SLN mapping data of 194 cases with ICG and MB were analyzed. The SLN identification rate was 99% (192/194) with ICG or ICG + MB, which was significantly higher than that with MB (92.8%,180/194) (*p* < 0.0001); the median SLN count was 3 (2) by ICG or ICG + MB, which was also higher than that by MB (2 (2)) (*p* < 0.0001); and the median metastatic SLN count was 1 (1.5) by ICG or ICG + MB, which was significantly higher than that by MB (1 (0)) (*p* = 0.014). The differences in sensitivity and FNR between ICG and MB were not prominent (*p* = 0.105). The results of fluorescence tracing were similar to those of combined tracing (Table 3, Supplement Fig.1B).

**Table 3:** The comparison of fluorescent tracing and methylene blue tracing

Parameters	N	ICG	MB	ICG+MB	*P*
SLN identification rate (%)	194	99.0 (192/194)	92.8 (180/194)	99.0% (192/194)	< 0.0001a
SLN counts (median (IQR))	194	3 (2)	2 (2)	3 (2)	< 0.0001c
False-negative rate (%)	37	0 (0/37)	8.1 (3/37)	0 (0/37)	0.105b
Sensitivity (%)	37	100 (37/37)	91.9 (34/37)	100 (37/37)	0.105b
Metastatic SLN rate (%)	194	19.1 (37/194)	17.5 (34/194)	19.1 (37/194)	0.903a
Metastatic SLN counts (median (IQR))	37	1 (1.5)	1 (0)	1 (1.5)	0.014c

*ICG* Indocyanine green, *MB* Methylene blue, *N* Number, *IQR* Interquartile range, *SLN* Sentinel lymph node. ^a^By Pearson’s chi-square test, ^b^by Fisher’s exactly test, ^c^by Kruskal-Wallis test

### SLNB with ICG was safe and reliable in both short-term and long-term evaluations

After the operation, follow-up was performed every 3–6 months within 5 years and every 12 months beyond 5 years. No anaphylactic reaction related to SLN tracers was found within 6 months, and the skin staining by ICG disappeared within 15 days when they were followed up for the first time after operation. Among the patients who underwent SLNB only (*N* = 157), 2 (SLN mapping by ICG + MB + RI in one patient, by ICG + MB in the other patient) developed lymphedema 6–8 months after the operation. After comprehensive treatment, the two patients were cured within a year.

**Fig. 3:**
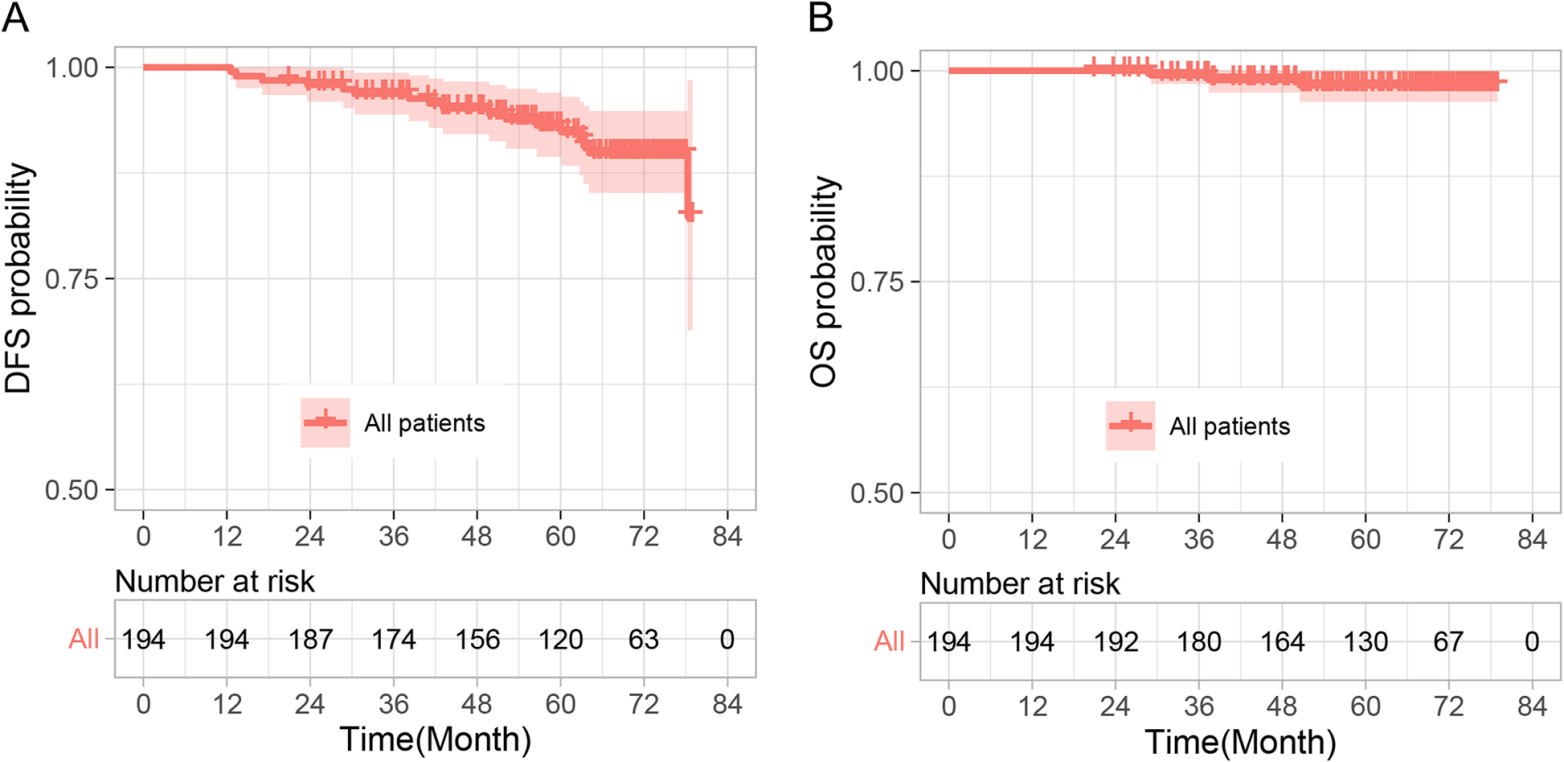
Survival analysis of all the 194 patients. **A** Disease-free survival (DFS). **B** Overall survival (OS). The shaded areas correspond to the 95% confidence bands

Over a median follow-up of 67 months, two patients of the 194 candidates experienced disease progression only in the ipsilateral armpit as the first site of treatment failure, and no disease progression was reported after ALND (Table 4). The 5-year disease-free survival rate and overall survival rate of this group were 92.3% and 98.3%, respectively (Fig. 3).

**Table 4:** Patients’ data of ipsilateral armpit as the only first site of treatment failure

	Molecular type	Age at diagnosis	DFS (month)	Treatment after recurrence	Outcome
Case 1	Luminal B	33	49.71	ALND	Living without disease progression
Case 2	TNBC	57	52.14	ALND	Living without disease progression

*ALND* Axillary lymph node dissection, *DFS* Disease-free survival, *TNBC* Triple-negative breast cancer

## Discussion

Although the dual mapping method with RI and BD is the gold standard for SLN identification, there are some difficulties in practice worldwide, including in China. In China, MB is the most commonly used tracer for SLN mapping, which is convenient and inexpensive. However, the lower detection rate and higher FNR of SLNs render this technique unsuitable for mapping SLNs alone [22]. After Kitai et al. reported the use of ICG as a fluorescent contrast to map lymphatic channels and SLNs, Guo J. et al. in China began to try SLN tracing with ICG [18, 23]. Compared with other tracers, SLN mapping with ICG is convenient, has good timeliness with a high detection rate, and does not require special sites. From June 2014 to September 2014, we began to use ICG as an SLN tracer. Consistent with the literature reports, ICG has the advantages of convenient use, low price, good timeliness, and a short learning curve. Each surgeon in our center mastered this technique through approximately 5 operations.

Compared with traditional tracers (RI, MB, or RI + MB), whether ICG can accurately evaluate SLNs has attracted extensive attention. It has been shown that the accuracy of ICG as an SLN tracer is superior to that of MB alone and is at least equivalent to RI or RI combined with MB [24, 25]. A few studies have compared the value of combined mapping of ICG and RI with or without patent blue for SLNB in breast cancer patients. The results indicated that there is no benefit of using patent blue for SLN mapping in breast cancer patients when using ICG and RI. ICG mapping outperformed patent blue in all patients [26]. When we established the learning curve in the first stage, we used three tracers to map the SLNs of the same patient. The results showed that the identification rate of SLNs with ICG or RI was similar to that with the dual technique (ICG + MB) or with the triad technique (ICG + MB + RI), and the identification rate of SLNs with MB was inferior to that with ICG, RI, or the combined techniques. At the end of 2015, 194 patients had undergone SLN mapping by ICG combined with MB. The data showed that fluorescence tracing was better than MB tracing in the identification rate of SLNs, and metastatic SLN counts.

Our data showed that the addition of MB combined mapping on the basis of the ICG fluorescence technique did not further increase the accuracy of SLNB. However, in clinical practice, we insist on using combined tracing because the staining of lymphatic vessels by MB is eye-catching, which can increase the convenience of operation.

An increasing number of studies have shown that ICG as an SLN tracer is superior to BD and similar to RI [26, 27]. The results of meta-analysis suggest that near-infrared fluorescence mapping using ICG could complement the RI method or provide an alternative in centers with poor accessibility to the RI technique [24, 25]. As a new SLN tracer, the short-term and long-term safety of ICG should be evaluated in the process of clinical application. Our results from all 194 patients showed that fluorescence tracing was more accurate than MB tracing, and ICG had no allergic reaction or skin tattoo effect in the short term. After a median follow-up of 67 months, only two patients experienced disease progression with the ipsilateral armpit as the first site of treatment failure, and the 5-year survival rate of DFS and OS was over 92%, indicating that the combined tracing technique of SLNs based on ICG has good long-term safety.

## Conclusion

Our retrospective study indicated that ICG as an SLN tracer is better than MB alone and comparable to RI + ICG + MB or ICG + MB. The short-term and long-term safety evaluation also showed that ICG can be used as a suitable supplement or alternative to traditional tracers to detect SLN in patients with early-stage breast cancer.

## Supplementary Information

**Additional file 1: Supplement Figure 1.** Accuracy of sentinel lymph node (SLN) tracing with different tracers and their combinations.

## Data Availability

All data generated or analyzed during this study are included in this published article.

## Abbreviations

ICG: Indocyanine green
SLNB: Sentinel lymph node biopsy
MB: Methylene blue
ALND: Axillary lymph node dissection
FNR: False-negative rate
BD: Blue dye
BCT: Breast-conserving therapy
HER-2: Human epidermal growth factor receptor 2
M: Mastectomy
MRM: Modified radical mastectomy
pTNM: Pathological tumor-node metastasis
IQR: Interquartile range
OS: Overall survival
RI: Technetium 99-labeled dextran
SLN: Sentinel lymph node
DFS: Disease-free survival
TNBC: Triple-negative breast cancer
