# Supplementary figures and images for: Short-term and long-term outcomes of indocyanine green for sentinel lymph node biopsy in early-stage breast cancer
Source: World J Surg Oncol. 2022 Aug 9;20:253. doi: 10.1186/s12957-022-02719-7 (PMC9361589; doi:10.1186/s12957-022-02719-7)

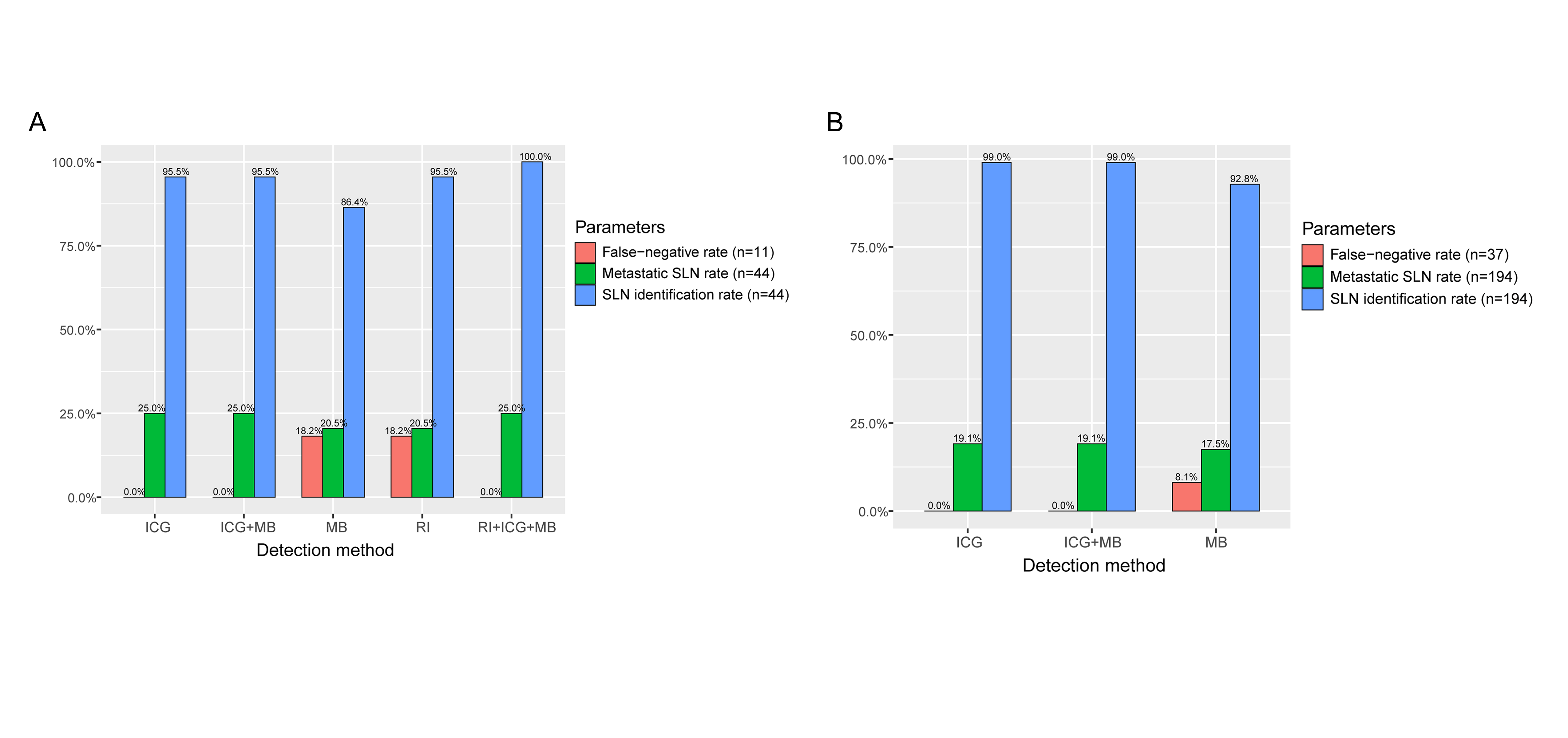

Supplement: Supplementary file 1 — Additional file 1: Supplement Figure 1. Accuracy of sentinel lymph node (SLN) tracing with different tracers and their combinations. [file 12957_2022_2719_MOESM1_ESM.tif]
